# Chinese herbal recipe versus diclofenac in symptomatic treatment of osteoarthritis of the knee: a randomized controlled trial [ISRCTN70292892]

**DOI:** 10.1186/1472-6882-4-19

**Published:** 2004-12-13

**Authors:** Supanimit Teekachunhatean, Puongtip Kunanusorn, Noppamas Rojanasthien, Kanit Sananpanich, Suwalee Pojchamarnwiputh, Sorasak Lhieochaiphunt, Sumalee Pruksakorn

**Affiliations:** 1 Department of Pharmacology, Faculty of Medicine, Chiang Mai University, Thailand; 2 Department of Orthopedics, Faculty of Medicine, Chiang Mai University, Thailand; 3 Department of Radiology, Faculty of Medicine, Chiang Mai University, Thailand; 4 Division of Pharmaceutical Sceinces, Faculty of Pharmacy, Chiang Mai University, Thailand; 5 Department of Microbiology, Faculty of Medicine, Chiang Mai University, Thailand

## Abstract

**Background:**

Duhuo Jisheng Wan (DJW) is perhaps the best known and most widely used Chinese herbal recipe for arthralgia, but the clinical study to verify its efficacy is lacking. The purpose of this study was to compare the efficacy of DJW versus diclofenac in symptomatic treatment of osteoarthritis (OA) of the knee.

**Methods:**

This study was a randomized, double-blind, double-dummy, controlled trial. The 200 patients suffering from OA of the knee, were randomized into the DJW and diclofenac group. The patients were evaluated after a run-in period of one week (week 0) and then weekly during 4 weeks of treatment. The clinical assessments included visual analog scale (VAS) score that assessed pain and stiffness, Lequesne's functional index, time for climbing up 10 steps, as well as physician's and patients' overall opinions on improvement.

**Results:**

Ninety four patients in each group completed the study. In the first few weeks of treatment, the mean changes in some variables (VAS, which assessed walking pain, standing pain and stiffness, as well as Lequesne's functional index) of the DJW group were significantly lower than those of the diclofenac group. Afterwards, these mean changes became no different throughout the study. Most of the physician's and patients' overall opinions on improvement at each time point did not significantly differ between the two groups. Approximately 30% of patients in both groups experienced mild adverse events.

**Conclusion:**

DJW demonstrates clinically comparable efficacy to diclofenac after 4 weeks of treatment. However, the slow onset of action as well as approximately equal rate of adverse events to diclofenac might limit its alternative role in treatment of OA of the knee.

## Background

Osteoarthritis (OA) is the most prevalent joint disorder characterized by articular cartilage degradation with an accompanying peri-articular bone response [1]. OA affects many joints, with diverse clinical patterns, but OA of the hip and knee is the major cause of disability [2]. A clinical manifestation of OA of the knee is pain in and around the joint that is typically worse with weight-bearing and at night. Other manifestations include morning stiffness, stiffness after rest, crepitation on motion, limited joint motion and/or joint deformity [3]. Although there are many treatment modalities, OA is still widely treated with nonsteroidal anti-inflammatory drugs (NSAIDs) [4]. Nonetheless, since the inflammatory component of OA is usually minimal, a need for the anti-inflammatory effect of NSAIDs used in this condition is still controversial [5-7]. Moreover, long-term use of NSAIDs is also directly related to many side effects, including gastrointestinal bleeding, hypertension, congestive heart failure, hyperkalemia, and renal insufficiency [8]. Although some of these disadvantages can be avoided by using paracetamol or selective cyclooxygenase II (COX-II) inhibitors, long-term use of paracetamol possibly leads to hepatotoxicity and chronic renal impairment [9,10]. In addition, the relatively high cost of selective COX-II inhibitors seems to be unsuitable for Thailand's present socio-economic status.

The use of Chinese and other foreign patent herbal medicines (pills and tablets) in arthralgia treatment is highly prevalent and increasing in Thailand, but importing these medicines from the People's Republic of China and other foreign countries is usually rather expensive. However, the cost of similar preparations can be minimized by using imported dried herbs available in Thailand as raw materials in the manufacturing process coupled with simple and inexpensive traditional drug manufacturing techniques. Thus, if clinical studies suggest that these herbal medicines are as effective and/or less toxic than conventional treatment, promotion of self-produced recipes in each community will lead to community-directed osteoarthritic treatment in Thailand.

The herbal recipe used in this study was "Duhuo Jisheng Wan (DJW)", which means pill of Pubescent angelica root and Mulberry mistletoe combination, and it was quoted from the book *Bei Ji Qian Jing Yao Fang *compiled by *Sun Simiao *in the *Tang *Dynasty (652 A.D.) [11,12]. Although this recipe is perhaps the best known and most widely used formula for arthralgia and also sold as a patent remedy [13], the clinical study to verify its efficacy (compared with conventional treatment) is lacking. Thus, the objectives of this study were to verify the efficacy of DJW and compare its efficacy versus diclofenac in symptomatic treatment of OA of the knee.

## Methods

### Research design

This randomized, double-blind, double-dummy, controlled trial was approved by the Medical Ethics Committee of the Faculty of Medicine, Chiang Mai University and was in compliance with the Helsinki Declaration.

### Subjects

Two hundred out-patients of either sex were recruited. They were aged over 40 years, and had been suffering from unilateral or bilateral OA of the knee according to the criteria of the American College of Rheumatology [3] for more than 3 months. After the use of usual medications had ceased for 7 days, the visual analog scale (VAS) score that assessed pain during the most painful knee movement had to be more than 40, and Lequesne's functional index [14] had to be over 7 points. Participants had to be able to walk and give both verbal and written information regarding the study. Signed informed consent was obtained prior to entry. Exclusion criteria included an underlying inflammatory arthropathy, hyperuricemia, expectation of surgery in the near future, recent injury in the area affected by OA of the knee, intra-articular corticosteroid injections within the last 3 months, hypersensitivity to NSAIDs, abnormal liver or kidney function tests, major abnormal finding on complete blood count, history of coagulopathies, history of peptic ulceration and upper GI hemorrhage, uncontrolled hypertension, congestive heart failure, hyperkalemia, pregnancy, lactation and malignant tumors.

### Treatment procedures

During a run-in period of 1 week (week 0), patients considered eligible for the study were informed to discontinue all analgesics, anti-inflammatory drugs, and other modalities for the treatment of arthralgia and arthritis. At the beginning of week 1, patients who still met the eligible criteria were randomized into 2 groups (DJW and diclofenac group) and treated for 4 weeks (Table 1). Other medications and treatment modalities for OA were prohibited during the study. In addition, a count of unused drugs and placebos was made weekly in order to check for the rates of compliance with medication.

**Table 1:** Treatment in the DJW and diclofenac group.

Treatment	DJW group	Diclofenac group
Capsule	Placebo	Diclofenac
Herbal capsule	DJW	Placebo

#### 1. Diclofenac and its placebo

Twenty five mg film-coated tablets of commercially marketed diclofenac sodium (Voltaren^®^) were provided by Novartis (Thailand) Co., Ltd. In order to completely blind the patients, each diclofenac tablet was packed into a capsule with an appearance identical to its placebo. Either diclofenac or placebo was prescribed at 1 capsule, 3 times a day, immediately after meals.

#### 2. DJW and its placebo

DJW and its placebo were prepared by the Department of Pharmaceutical Sciences, Faculty of Pharmacy, Chiang Mai University. It consisted of 7.75% each of Radix Angelicae Pubescentis (Duhuo), Radix Gentianae Macrophyllae (Qinjiao), Cortex Eucommiae (Duzhong), Radix Achyranthis Bidentatae (Niuxi), Radix Angelicae Sinensis (Danggui), Herba Taxilli (Sangjisheng), Radix Rehmanniae Preparata (Shudihuang), Rhizoma Chuanxiong (Chuanxiong), Cortex Cinnamomi (Rougui) and Radix Ledebouriellae (Fangfeng), 5% each of Radix Paeoniae Alba (Baishao), Radix Codonopsis (Dangshen), Radix Glycyrrhizae (Gancao) and Poria (Fuling), as well as 2.5% of Herba Asari (Xixin).

Xixin, Niuxi, Shudihuang and Rougui were imported from the Shantou Traditional Chinese Medicine Factory, the People's Republic of China (PRC). The remaining herbs were imported from the Qixin Co., Ltd. (Hebei Province), PRC. Each pulverized ingredient was mixed thoroughly together according to the formula mentioned above and prepared into honeyed pills, which were baked in a hot air oven until completely dry, and then pulverized. The pulverized powder was finally filled into capsules of 500 mg per capsule. The quality control and standardization of DJW (i.e., assessment of weight variation, disintegration time, screening for microorganisms and aflatoxin) were conducted by using guidelines recommended by the Food and Drug Administration of Thailand [15]. DJW and its placebo were prepared in 4 separate lots. Every lot had to pass for quality control and standardization before prescription and they were used within 8 weeks in order to ascertain the stability of active substances, and avoid microorganism and aflatoxin contamination during the study. DJW was prescribed at 6 capsules (3 g) each time, 3 times a day, immediately after meals. Its placebo, with identical appearance, was made from cane sugar and prescribed at the same dosage as the DJW.

### Assessments

Clinical assessments were evaluated for base-line data at the end of a run-in period (week 0) and then weekly for 4 weeks. These assessments included 100-mm VAS that assessed pain (classified into walking pain, standing pain, pain during climbing up and down stairs, night pain, resting pain, total pain, pain during the most painful knee movement), 100-mm VAS that assessed stiffness (classified into morning stiffness, stiffness after rest and total stiffness), Lequesne's functional index that assessed the patient's daily activities (score ranging from 0–24) [14], and time for climbing up 10 steps. The participants self-rated the VAS and Lequesne's functional index, and they were allowed to view their own previously recorded scores.

At the end of week 1–4, 100-mm VAS that assessed the physician's and patients' overall opinions on improvement were also evaluated. The assessment forms were designed so that the patients and evaluator could view their own previously recorded scores, but they were not allowed to view each other's VAS. Clinical assessments were evaluated by the same physician who had been blinded to the treatment. Complete physical examination and non-directive questioning for adverse events were also performed weekly for 4 weeks in order to acquire a safety assessment.

### Statistical analysis

In within the group analysis, the mean VAS and Lequesne's functional index between base-line and the following weeks were compared by a non-parametric Wilcoxon's signed-rank test, whereas, the average time for climbing up 10 steps was compared by the paired t-test.

In the analysis between the groups, a non-parametric Wilcoxon's rank-sum test was used to determine whether the two groups differed in the physician's and patients' overall opinions on improvement. In addition, the mean changes in VAS that assessed pain and stiffness, as well as Lequesne's functional index were compared by the same test. The student's t-test was used to compare the mean changes in the time for climbing up 10 steps.

## Results

**Figure 1:**
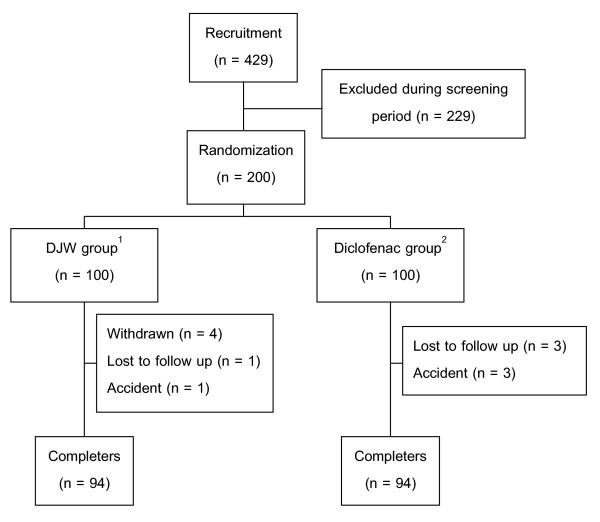
Flow chart of patients who participated in the clinical trial. ^1^DJW group received DJW plus placebo of diclofenac. ^2^Diclofenac group received diclofenac plus placebo of DJW.

A total of 429 patients were recruited into this study, of whom 229 were excluded (Figure 1). The remaining 200 patients were randomized into the DJW and diclofenac group, 100 patients per group. In the DJW group, 4 patients withdrew from the study due to ineffectiveness (n = 3) and transportation problem (n = 1), 1 patient was lost to follow up and another one had a traffic accident during the study. In the diclofenac group, 3 patients were lost to follow up and 3 were withdrawn due to accidents. Thus, each group comprised 94 completers. The two treatment groups were not significantly different in demographic data e.g., sex, age, weight, height, duration of OA, location of OA (Table 2) and base-line data for the major outcome assessment (VAS, Lequesne's functional index and time for climbing up 10 steps). The radiographic findings at entry (Table 3) were not different between both groups. During the study, the rates of compliance with medication in the DJW group were 94%, whereas, those in the diclofenac group were 96%. Since few patients withdrew from the trial, the results were not substantially affected, whether the statistical method was performed by an intention to treat (ITT) analysis or an analysis on available completers. Thus, the following data showed the findings from the ITT analysis.

**Table 2:** Demographic data and base-line data for the major outcome assessments of participants evaluated at the end of a run-in period (week 0).

	Treatment groups		
		
Characteristics	DJW	Diclofenac	*p *value
n (M:F)	100 (22:78)	100 (19:81)	NS
Age (y)*	62.66 (9.46)	62.38 (8.22)	NS
Body weight (kg)*	60.47 (10.34)	60.13 (10.89)	NS
Height (m)*	1.51 (0.07)	1.51 (0.07)	NS
BMI (kg/m^2^)*	26.52 (4.38)	26.35 (3.85)	NS
Duration of OA (y)*	5.46 (5.48)	4.79 (4.24)	NS
Localization of OA			NS
Right knee	17	17	
Left knee	14	14	
Both knees	69	69	
VAS the assessed pain (mm)*			
Walking pain	64.53 (24.92)	64.78 (25.14)	NS
Standing pain	52.42 (25.87)	53.52 (24.69)	NS
Pain during climbing up and down stairs	63.08 (20.87)	62.69 (23.21)	NS
Night pain	50.15 (26.74)	48.45 (28.18)	NS
Resting pain	38.48 (22.09)	37.12 (26.08)	NS
Total pain^a^	268.65 (88.87)	266.55 (89.33)	NS
Pain during the most painful knee movement	82.25 (16.15)	81.17 (16.56)	NS
VAS that assessed stiffness (mm)*			
Morning stiffness	53.53 (27.38)	58.32 (26.40)	NS
Stiffness after rest	68.52 (22.76)	70.45 (22.32)	NS
Total stiffness^b^	122.05 (41.98)	128.76 (42.34)	NS
Lequesne's functional index*	14.20 (3.13)	14.80 (2.61)	NS
Time for climbing up 10 steps*	13.44 (4.85)	13.32 (5.10)	NS

*Data represent mean (SD). ^a^Summation of VAS that assessed walking pain, standing pain, pain during climbing up and down stairs, night pain and resting pain. ^b^Summation of VAS that assessed morning stiffness and stiffness after rest. NS: no statistical significance.

**Table 3:** The radiographic findings at entry into the study.

	Treatment groups		
		
Radiographic findings	DJW (169 knees)	Diclofenac (169 knees)	*p *value
Kellgren and Lawrence X-ray grade [20]			NS
Grade 2	31	23	
Grade 3	71	80	
Grade 4	67	66	
Knee compartment with most severe changes			NS
Medial tibiofemoral	131	135	
Lateral tibiofemoral	16	8	
Patellofemoral	22	26	

The VAS that assessed pain and stiffness at the end of week 1–4 decreased significantly when compared to their own base-line values (within the group analysis), as did Lequesne's functional index and time for climbing up 10 steps (Table 4). At the end of week 4, the percentages of improvement in VAS that assessed pain and stiffness were higher than 65% in both groups, whereas, the percentages of improvement in Lequesne's functional index and time for climbing up 10 steps were approximately 40% and 20%, respectively.

**Table 4:** Mean VAS that assessed pain and stiffness, Lequesne's functional index and time for climbing up 10 steps in intent-to-treat patients (n = 100/group).

Variable	Treatment Group	Week 0	Week 1	Week 2	Week 3	Week 4	% improvement^a^
VAS that assessed pain (mm)							
Walking pain	DJW	64.53 (24.92)	47.58* (25.33)	37.72* (25.00)	28.00* (23.25)	18.06* (20.76)	72.01
	Diclofenac	64.78 (25.14)	44.08* (23.43)	34.99* (22.07)	24.21* (21.00)	14.31* (16.10)	77.91
Standing pain	DJW	52.42 (25.87)	39.81* (26.09)	31.61* (24.89)	24.29* (22.98)	16.89* (20.59)	67.78
	Diclofenac	53.52 (24.69)	37.60* (24.06)	28.19* (22.40)	21.12* (21.16)	12.86* (16.69)	75.97
Pain during climbing up and down stairs	DJW	63.08 (20.87)	46.31* (26.56)	36.40* (25.67)	28.16* (24.03)	18.41* (21.50)	70.81
	Diclofenac	62.69 (23.21)	43.90* (22.29)	32.61* (22.42)	24.59* (21.79)	15.83* (19.65)	74.75
Night pain	DJW	50.15 (26.74)	33.44* (27.27)	23.56* (22.79)	15.68* (18.14)	9.27* (15.04)	81.52
	Diclofenac	48.45 (28.18)	28.93* (22.82)	20.87* (19.56)	15.02* (17.87)	8.65* (14.68)	82.15
Resting pain	DJW	38.48 (22.09)	27.25* (21.99)	19.96* (19.98)	12.64* (15.56)	7.42* (13.09)	80.72
	Diclofenac	37.12 (26.08)	22.84* (20.62)	16.26* (18.19)	11.30* (16.40)	6.58* (13.96)	82.27
Total pain^b^	DJW	268.65 (88.87)	194.38* (105.06)	149.24* (103.19)	108.76* (92.54)	70.04* (83.94)	73.93
	Diclofenac	266.55 (89.33)	177.34* (85.49)	132.91* (84.50)	96.21* (81.94)	58.23* (70.43)	78.15
Pain during the most painful knee movement	DJW	82.25 (16.15)	63.31* (26.35)	49.77* (28.70)	37.69* (28.45)	26.81* (27.70)	67.40
	Diclofenac	81.17 (16.56)	56.79* (24.87)	43.64* (27.30)	33.10* (27.17)	22.84* (25.85)	71.86
VAS that assessed stiffness (mm)							
Morning stiffness	DJW	53.53 (27.38)	36.61* (25.56)	28.04* (23.86)	19.66* (20.53)	12.34* (17.69)	76.95
	Diclofenac	58.32 (26.40)	38.73* (23.87)	28.52* (21.93)	20.19* (20.23)	12.90* (17.34)	77.88
Stiffness after rest	DJW	68.52 (22.76)	51.69* (24.93)	39.40* (25.31)	29.05* (24.60)	19.62* (23.06)	71.37
	Diclofenac	70.45 (22.32)	49.71* (24.68)	39.54* (24.97)	28.23* (24.17)	18.90* (20.60)	73.17
Total stiffness^c^	DJW	122.05 (41.98)	88.30* (45.93)	67.44* (46.25)	48.71* (42.82)	31.96* (38.84)	73.81
	Diclofenac	128.76 (42.34)	88.44* (43.84)	68.06* (43.03)	48.42* (41.97)	31.80* (36.07)	75.30
Lequesne's functional index (score)	DJW	14.20 (3.13)	11.60* (4.11)	11.05* (4.04)	9.93* (4.40)	8.92* (4.60)	37.18
	Diclofenac	14.80 (2.61)	10.89* (3.38)	10.65* (3.55)	9.59* (3.52)	8.64* (3.83)	41.62
Time for climbing up 10 steps (s)	DJW	13.44 (4.85)	11.65* (4.75)	11.42* (4.67)	10.94* (4.73)	10.50* (4.38)	21.88
	Diclofenac	13.32 (5.10)	11.26* (5.12)	11.14* (5.72)	10.61* (5.51)	10.18* (4.46)	23.57

Data represent mean (SD). ^a^Calculated by (mean_week0_-mean_week4_) × 100/mean_week0_. ^b^Summation of VAS that assessed walking pain, standing pain, pain during climbing up and down stairs, night pain and resting pain. ^c^Summation of VAS that assessed morning stiffness and stiffness after rest. *p < 0.05 versus base-line value.

When the statistical analysis between groups was performed, the mean changes in VAS that assessed pain during climbing up and down the stairs, night pain, resting pain, total pain, and time for climbing up 10 steps did not differ significantly between both groups (Table 5). Nonetheless, the mean changes in VAS that assessed walking pain, standing pain, and stiffness were significantly different during week 0–1, whereas, differences in mean changes in Lequesne's functional index were found during week 0–1 and 0–2. Afterwards, the mean changes in these variables became no different throughout the study.

**Table 5:** Mean changes of VAS that assessed pain and stiffness, Lequesne's functional index and time for climbing up 10 steps in intent-to-treat patients (n = 100/group).

Variable	Treatment Group	Week 0–1	Week 0–2	Week 0–3	Week 0–4
VAS that assessed pain (mm)					
Walking pain	DJW	-16.96 (1.68)	-26.82 (1.97)	-36.54 (2.31)	-46.48 (2.41)
	Diclofenac	-20.70^† ^(1.60)	-29.80 (1.95)	-40.58 (2.26)	-50.47 (2.38)
Standing pain	DJW	-12.61 (1.80)	-20.81 (2.23)	-28.13 (2.28)	-35.53 (2.34)
	Diclofenac	-15.93^† ^(1.33)	-25.33 (1.83)	-32.41 (2.04)	-40.66 (2.25)
Pain during climbing up and down stairs	DJW	-16.78 (1.95)	-26.68 (2.30)	-34.93 (2.26)	-44.67 (2.22)
	Diclofenac	-18.79 (1.40)	-30.08 (1.91)	-38.11 (2.04)	-46.86 (2.35)
Night pain	DJW	-16.71 (2.32)	-26.60 (2.25)	-34.47 (2.42)	-40.88 (2.59)
	Diclofenac	-19.52 (1.98)	-27.58 (2.30)	-33.43 (2.51)	-39.80 (2.81)
Resting pain	DJW	-11.23 (1.24)	-18.52 (1.46)	-25.84 (1.86)	-31.06 (2.02)
	Diclofenac	-14.28 (1.34)	-20.86 (1.91)	-25.82 (2.07)	-30.54 (2.38)
Total pain^a^	DJW	-74.27 (6.53)	-119.42 (7.42)	-159.90 (7.85)	-198.61 (8.51)
	Diclofenac	-89.21 (5.25)	-133.64 (7.02)	-170.34 (7.65)	-208.33 (9.03)
Pain during the most painful knee movement	DJW	-18.94 (2.11)	-32.48 (2.63)	-44.56 (2.77)	-55.44 (2.67)
	Diclofenac	-24.38 (2.10)	-37.53 (2.51)	-48.07 (2.57)	-58.33 (2.59)
VAS that assessed stiffness (mm)					
Morning stiffness	DJW	-16.93 (1.98)	-25.50 (2.24)	-33.87 (2.46)	-41.19 (2.58)
	Diclofenac	-19.59^† ^(1.69)	-29.80 (2.10)	-38.13 (2.47)	-45.42 (2.63)
Stiffness after rest	DJW	-16.83 (1.97)	-29.12 (2.41)	-39.48 (2.50)	-48.91 (2.54)
	Diclofenac	-20.74^† ^(1.72)	-30.91 (2.07)	-42.22 (2.29)	-51.55 (2.40)
Total stiffness^b^	DJW	-33.76 (3.48)	-54.62 (4.02)	-73.35 (4.21)	-90.10 (4.27)
	Diclofenac	-40.33^† ^(3.05)	-60.71 (3.72)	-80.35 (4.21)	-96.97 (4.47)
Lequesne's functional index (score)	DJW	-2.60 (0.34)	-3.15 (0.32)	-4.28 (0.37)	-5.29 (0.38)
	Diclofenac	-3.92^† ^(0.31)	-4.16^† ^(0.32)	-5.22 (0.36)	-6.16 (0.40)
Time for climbing up 10 steps (s)	DJW	-1.79 (0.33)	-2.02 (0.31)	-2.50 (0.32)	-2.94 (0.32)
	Diclofenac	-2.05 (0.31)	-2.18 (0.34)	-2.71 (0.34)	-3.13 (0.33)

Data represent mean (SD). ^a^Summation of VAS that assessed walking pain, standing pain, pain during climbing up and down stairs, night pain and resting pain. ^b^Summation of VAS that assessed morning stiffness and stiffness after rest. ^†^*p *< 0.05 versus the DJW group at the same duration of treatment.

The physician's and patients' overall opinions on improvement, as measured on VAS, are shown in Table 6. The physician's overall opinion on improvement at each time point did not significantly differ between the two groups. However, differences between groups (DJW versus diclofenac group) were found in the patients' overall opinion at week 1 (32.58 ± 23.18 versus 37.48 ± 18.59), but no differences were demonstrated at the remaining time-points.

**Table 6:** VAS that assessed physician's and patients' overall opinions on improvement^a ^during treatment (intent-to-treat data set).

Variable	Treatment group	n	Week 1	Week 2	Week 3	Week 4
Physician's overall opinion	DJW	98^b^	56.69 (11.32)	57.30 (11.32)	60.06 (12.47)	62.55 (11.67)
	Diclofenac	97^b^	59.84 (7.53)	59.63 (7.74)	62.11 (7.57)	63.35 (7.90)
Patients' overall opinion	DJW	98^b^	32.58 (23.18)	45.53 (24.74)	58.10 (26.84)	71.13 (24.68)
	Diclofenac	97^b^	37.48* (18.59)	50.24 (18.79)	62.88 (19.75)	75.30 (17.95)

Data represent mean (SD). ^a^0 = no improvement, 100 = best possible improvement. ^b^2 patients in the DJW group and 3 patients in the diclofenac group could not be assessed due to loss to follow up or withdrawal during week 0. *p < 0.05 versus the DJW group at the corresponding week.

The majority of patients in both groups experienced no adverse events (72% vs. 73% for DJW and diclofenac groups, respectively). All adverse events reported were mild in intensity in both groups. The most common adverse events occurring in the DJW and diclofenac group were raised blood pressure (16% vs. 19%), central nervous system symptoms including dizziness, somnolence and drowsiness (16% vs. 11%), and gastrointestinal symptoms including nausea/vomiting, dyspepsia, diarrhea and constipation (12% vs. 5%). The least common adverse events were increased appetite, cramp, rash and flu. More than one adverse events might be occurred in some patients. However, the percentages of patients who experienced each adverse event in both groups were not significantly different.

In summary, the VAS that assessed pain and stiffness, Lequesne's functional index and time for climbing up 10 steps at each time point decreased significantly in the DJW and diclofenac group when compared to their own base-line values. The mean changes in all VAS that assessed pain, except those for walking and standing, did not differ significantly between both groups. The differences in mean changes in the VAS that assessed walking pain, standing pain and stiffness were found only during week 0–1, whereas, those in Lequesne's functional index were found during week 0–1 and 0–2.

## Discussion

Since the preparations and dosages of DJW and diclofenac were different, this study was designed as a randomized, double dummy, controlled trial in order to completely blind both patients and physician (double-blind). Therefore, the placebo of DJW was also prescribed for the patients in the diclofenac group, and vice versa, the placebo of diclofenac was prescribed for the patients in the DJW group.

Among the 15 herbs used as raw materials in DJW, Xixin (Herba Asari) seemed to be the most toxic, due to its pungent taste and warm property [16]. Generally, a large dose of this herb is not recommended in a tropical country (such as Thailand) because of the potential aggravation of internal heat. Thus, the amount of Xixin in the DJW recipe used in this study was reduced from 7.75% to 2.5%.

In an ITT analysis (and analysis on completers), the mean changes in some variables between the two groups were significantly different after the first few weeks of treatment, and became no different afterwards. These differences suggest that the onset of DJW is significantly slower than diclofenac for at least 2 weeks (with respect to walking pain, standing pain, morning stiffness, stiffness after rest, total stiffness and patients' overall opinion) or 3 weeks (with respect to Lequesne's functional index). The reason why DJW needs a few weeks to exert its effect may be due to 3 possibilities. Firstly, from the pharmacokinetic point of view, the elimination half-life of the active ingredients in DJW might be too long, and therefore needs weeks to accumulate until a steady state concentration is reached (normally 4–5 half-lives) and its maximal therapeutic effect is evident. Secondly, from the pharmacodynamic point of view, DJW may exert its effects via several probable mechanisms (similar to many novel biologic treatments of arthropathy) involved modifications of cartilage metabolism, normalized viscosity and elasticity of synovial fluid, etc. These mechanisms of action might resemble many symptomatic slow acting drugs in osteoarthritis (SYSADOA) such as glucosamine sulfate, intra-artricular hyaluronan, and others. These interventions always need a period of time to exert their therapeutic action. Thirdly, the major effect of DJW might be the result from placebo effect and/or natural fluctuation of the OA symptoms. It could be simply that diclofenac worked quickly, but patients in both groups got better anyway by 2–3 weeks. Although the last possibility cannot be entirely ruled out, but it seems unlikely because even there is a tendency of OA symptoms to improve after placebo treatment, it has been reported that diclofenac was significantly superior to placebo in relieving pain, improving stiffness, and improving physical function after 4 weeks of treatment [17]. Furthermore, we also found that oral administration of the ethanol extract of DJW possessed both central and peripheral analgesic activities in animal model, even when the DJW extract was given in the equivalent dose used in human (mg/kg of human dose corrected by intra- and inter-specie variations) [to be published data]. In clinical practice, this slower onset of action and probable need for rescue analgesics (e.g., paracetamol as needed) during the first 2–3 weeks after initiation of DJW should be the important limitations of using DJW as an alternative treatment for OA of the knee. Moreover, the patient's compliance with such a high dosage of DJW (9 g/day or 18 capsules/day) is an important issue to be concerned.

Since this study demonstrated that approximately 30% of study subjects in each group experienced adverse events, this data suggest that the toxicity profiles of DJW are similar to diclofenac. Therefore, cautious use of DJW should be considered in the same manner as using diclofenac including other NSAIDs. However, the gastrointestinal adverse effects in the diclofenac group were quite low when compared to other short-term NSAIDs studies [18,19]. This might be due to the exclusion of patients with a high risk of adverse effects from NSAIDs during the screening visit. Since the relief of joint pain afforded by paracetamol is comparable with that achievable with NSAIDs, paracetamol merits a trial as initial therapy, based on its overall cost, efficacy, and toxicity profile [3,7]. In this circumstance, the rather high rate of adverse events from DJW should be another limitation of using DJW as an alternative, especially to paracetamol, in symptomatic treatment of OA of the knee.

## Conclusion

DJW demonstrates clinically comparable efficacy to diclofenac after 4 weeks of treatment. However, the slow onset of action as well as approximately equal rate of adverse events to diclofenac might limit its alternative role in treatment of OA of the knee.

## Competing interests

The author(s) declare that they have no competing interests.

## Authors' contributions

ST carried out the randomization, supervised data collection and analysis, and drafted the manuscript. PK participated in the design of the study and performed the statistical analysis. NR participated in the selection of patients eligible for the study. KS carried out the outcome assessments. SP participated in the report of radiographic findings of knee. SL participated in the preparation of DJW and its placebo. SP carried out the screening for microorganism contamination in DJW and its placebo. All authors read and approved the final manuscript.

## Pre-publication history

The pre-publication history for this paper can be accessed here:



## References

[B1] Felson DT (2004). An update on the pathogenesis and epidemiology of osteoarthritis. Radiol Clin North Am.

[B2] Drury PL, Shipley M, Kumar P, Clark M (1998). Rheumatology and bone disease. In Clinical medicine.

[B3] Hochberg MC, Altman RD, Brandt KD, Clark BM, Dieppe PA, Griffin MR, Moskowitz RW, Schnitzer TJ (1995). Guidelines for the medical management of osteoarthritis. Part II. Osteoarthritis of the knee. American College of Rheumatology. Arthritis Rheum.

[B4] Hungin AP, Kean WF (2001). Nonsteroidal anti-inflammatory drugs: overused or underused in osteoarthritis?. Am J Med.

[B5] Wegman A, van der Windt D, van Tulder M, Stalman W, de Vries T (2004). Nonsteroidal antiinflammatory drugs or acetaminophen for osteoarthritis of the hip or knee? A systematic review of evidence and guidelines. J Rheumatol.

[B6] Brooks PM, Potter SR, Buchanan WW (1982). NSAIDs and osteoarthritis-help or hindrance?. J Rheumatol.

[B7] Bradley JD, Brandt KD, Katz BP, Kalasinski LA, Ryan SI (1991). Comparison of an antiinflammatory dose of ibuprofen, an analgesic dose of ibuprofen, and acetaminophen in the treatment of patients with osteoarthritis of the knee. N Engl J Med.

[B8] Buffum M, Buffum JC (2000). Nonsteroidal anti-inflammatory drugs in the elderly. Pain Manag Nurs.

[B9] Bolesta S, Haber SL (2002). Hepatotoxicity associated with chronic acetaminophen administration in patients without risk factors. Ann Pharmacother.

[B10] McLaughlin JK, Lipworth L, Chow WH, Blot WJ (1998). Analgesic use and chronic renal failure: a critical review of the epidemiologic literature. Kidney Int.

[B11] Xu XC, Yuan JR, Tian JZ, Chen SM (1994). Commonly used Chinese patent medicines.

[B12] Geng JY, Huang WQ, Ren TC, Ma XF (1997). Practical traditional Chinese medicine & pharmacology: Herbal Formulas.

[B13] Analysis of Chinese herb prescriptions for rheumatoid arthritis. http://www.itmonline.org/arts/arthritis.htm.

[B14] Lequesne MG, Mery C, Samson M, Gerard P (1987). Indexes of severity for osteoarthritis of the hip and knee validation-value in comparison with other assessment tests. Scand J Rheumatology.

[B15] Thai Food and Drug Admistration, Ministry of Public Health (2000). Handbook of herbal products for community-directed economy.

[B16] Asarum herb (Xixin). http://www.johnlewconsultancy.com/headache/f-herb/hb-baizhi.html.

[B17] Schnitzer TJ, Beier J, Geusens P, Hasler P, Patel SK, Senftleber I, Gitton X, Moore A, Sloan VS, Poor G (2004). Efficacy and safety of four doses of lumiracoxib versus diclofenac in patients with knee or hip primary osteoarthritis: a phase II, four-week, multicenter, randomized, double-blind, placebo-controlled trial. Arthritis Rheum.

[B18] Valat JP, Accardo S, Reginster JY, Wouters M, Hettich M, Lieu PL (2001). A comparison of the efficacy and tolerability of meloxicam and diclofenac in the treatment of patients with osteoarthritis of the lumbar spine. Inflamm Res.

[B19] Gagnier P (1993). Review of the safety of diclofenac/misoprostol. Drug.

[B20] Kellgren JH, Lawrence JS (1957). Radiological assessment of osteoarthritis. Ann Rheum Dis.

